# Interpretations on Chat Generative Pre-training Transformer vs. surgeons on pancreatic cancer queries: accuracy and empathy evaluated by patients and experts

**DOI:** 10.1590/1806-9282.20250500

**Published:** 2025-09-19

**Authors:** Ilker Sengul, Demet Sengul

**Affiliations:** 1 Giresun University, Faculty of Medicine, Division of Endocrine Surgery — Giresun, Turkey.; 2 Giresun University, Faculty of Medicine, Department of General Surgery — Giresun, Turkey.; 3 Giresun University, Faculty of Medicine, Department of Pathology — Giresun, Turkey.

Dear Editor,

Felix, qui potuit rerum cognoscere causas. Hepatobiliopancreatic disorders^1,2^, per se, are a crucial discipline that may demand a gracious approach in human beings regarding disorders and therapeutic options, to date. We have read with a great deal of interest the article by Klotz et al.^3^, entitled "ChatGPT vs. surgeons on pancreatic cancer queries: accuracy & empathy evaluated by patients and experts." This utilitarian research seems to demand a determination of the efficacy of the artificial intelligence (AI), namely ChatGPT, in enhancing patient—provider interactions. For this purpose, 24 cases and 25 surgeons had been enrolled, from Jun 23 to Jul 21, 2023, in this original study, which provides a promising audit of the potential of AI to respond to medical queries in a perceptive and empathic manner. As such, pancreatic cancer surgery allows fine AI to translate technical data into comprehensible denouements and solutions for the cases. ChatGPT's interpretations were well received, regarding comprehension, and might lead to an AI tool that effectively bridges the gap between healthcare professionals and patients. However, would the utilization of a more multifarious group of experts and other AI systems, instead of a blinded comparison of ChatGPT by two surgeons, alter the outcome(s) of this study? Furthermore, would a broader, more assorted, miscellaneous group of patients and healthcare providers, instead of the mentioned sample size, move on this work's denouement(s)? This issue merits further investigation. We thank Klotz et al.^3^ for their valuable study on pancreatic cancer of hepatobiliopancreatic surgery.

## Data Availability

The datasets generated and/or analyzed during the current study are available from the corresponding author upon reasonable request.
